# A Sting Beyond the Skin: Jellyfish Envenomation Presenting With Paralytic Ileus and Acute Urinary Retention

**DOI:** 10.7759/cureus.82281

**Published:** 2025-04-15

**Authors:** Kajananan Sivagurunathan, Nalayini Jegathesan

**Affiliations:** 1 Internal Medicine, District General Hospital Kilinochchi, Kilinochchi, LKA; 2 Internal Medicine, Teaching Hospital Jaffna, Jaffna, LKA

**Keywords:** acute urinary retention (aur), environmental toxicology, jellyfish, jellyfish sting, marine toxin, paralytic ileus

## Abstract

Jellyfish envenomation typically causes localized pain and systemic reactions, but rare complications such as acute urinary retention and paralytic ileus can occur. We report a case of a 21-year-old fisherman from northern Sri Lanka who developed urinary retention and paralytic ileus following a jellyfish sting. He initially experienced severe pain and itching, followed by acute urinary retention, progressive abdominal distension, vomiting, and absent bowel opening. Imaging confirmed paralytic ileus without mechanical obstruction. The patient was managed conservatively with catheterization, bowel rest, intravenous fluids, analgesia, and nasogastric decompression, leading to full recovery within 48 hours. Although the exact mechanism remains unclear, previous studies have hypothesized that jellyfish neurotoxins may affect autonomic regulation. This study highlights the need for awareness of rare urological and gastrointestinal complications following jellyfish stings and emphasizes the importance of timely supportive management.

## Introduction

Jellyfish envenomation is a common marine hazard, particularly in coastal areas. Jellyfish, belonging to the phylum Cnidaria, are invertebrates found in saltwater and brackish environments. They have a bell-shaped body and long, detachable tentacles containing nematocysts, which inject venom into their prey. While they do not actively seek humans, accidental stings occur when people come into contact with their tentacles. Approximately 100 of the 10,000 jellyfish species are harmful to humans, with around 150 million stings reported annually worldwide [1]. In the northern part of Sri Lanka, most of the victims are fishermen [2]. The venom, released through barbed tubes from the nematocysts, can cause a range of clinical symptoms, from localized pain and erythema to severe systemic reactions such as anaphylaxis and cardiovascular collapse.

Most reported cases of jellyfish stings result in immediate cutaneous or systemic effects such as pain, erythema, or anaphylaxis. However, gastrointestinal and urological involvement, such as paralytic ileus and urinary retention, is rarely documented in the literature. The mechanisms of particular complications remain poorly understood. This study presents a rare occurrence of paralytic ileus and acute urinary retention following a jellyfish sting in a young, healthy male. This report aimed to highlight these unusual complications and provide insight into their clinical management.

## Case presentation

A 21-year-old previously healthy male fisherman sustained a jellyfish sting while fishing in the coastal waters of Pallikuda, Kilinochchi, Sri Lanka. The exact species of jellyfish responsible for the sting could not be identified. Immediately after the sting, he experienced severe burning pain and itching at the sting site on the right side of his face, followed by intense generalized body pain. Upon presentation to the hospital two hours later, he was in severe pain. His vital parameters were as follows: blood pressure of 140/90 mmHg, pulse rate of 108 bpm, respiratory rate of 20 breaths per minute, and oxygen saturation of 98% on room air. There were no signs of anaphylaxis or respiratory distress at the time. Additionally, no significant erythema or swelling at the sting site was observed. He was symptomatically managed with analgesics and antihistamines, including regular paracetamol 1 g every 6 hours, subcutaneous morphine 5 mg as needed, intravenous chlorphenamine 4 mg every 12 hours, and ranitidine 50 mg every 12 hours. He was also adequately hydrated with normal saline. Laboratory investigations, including serum creatinine, liver enzymes, and serum electrolytes, were all within normal limits except increased leucocyte count (Table 1). The leukocytosis was consistent with the physiological response to severe stress following sting.

**Table 1:** Summary of the laboratory test results of the patient.

Laboratory test	Result	Normal range
White cell count	19.32 × 10^9^/L	4.0-10.0 × 10^9^/L
Hemoglobin	12.8 g/dL	12-15 g/dL
Platelets	265 × 10^9^/L	150-400 × 10^9^/L
C-reactive protein	0.1 mg/L	0-10 mg/L
Aspartate transferase	24.7 U/L	15-37 U/L
Alanine aminotransferase	16.5 U/L	16-63 U/L
Sodium	141.6 mmol/L	136-145 mmol/L
Potassium	3.56 mmol/L	3.5-5.1 mmol/L
Creatinine	0.86 mg/dL	0.7-1.3 mg/dL

**Figure 1:**
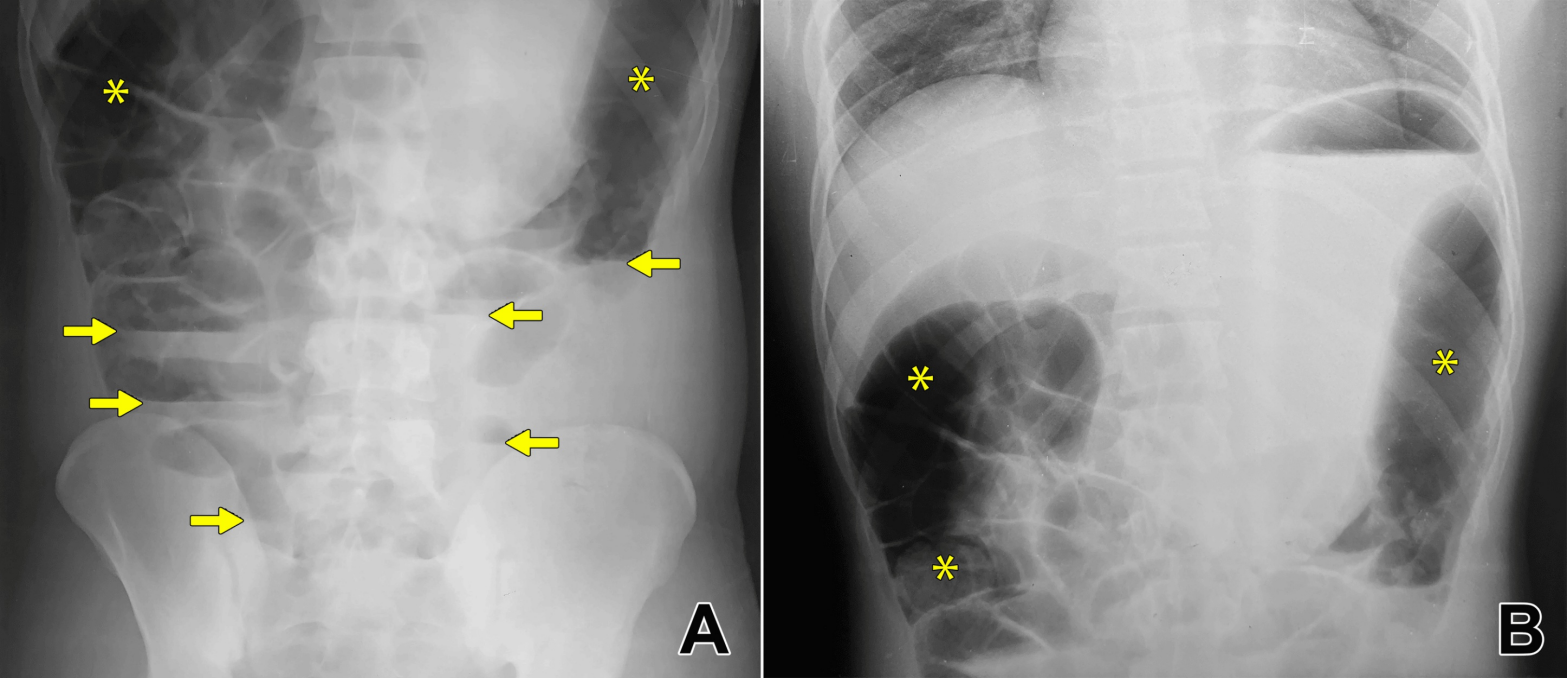
Abdominal X-rays showing dilated bowel loops with air-fluid levels. (A) Erect abdominal X-ray shows multiple dilated bowel loops (asterisk) with air-fluid levels (arrow); (B) supine abdominal X-ray demonstrates diffusely dilated bowel loops (asterisk) without a clear transition point.

On the following morning, he experienced difficulty in urination and developed acute urinary retention, with examination findings of palpable bladder, necessitating catheterization. The diagnosis of acute urinary retention was made clinically based on a distended bladder on examination. Imaging studies or post-void residual measurements were not performed prior to catheterization. On the same night, he developed progressive abdominal distension, pain (not colicky), nausea, and multiple episodes of vomiting. He also experienced absence of bowel movements and an inability to pass flatus or stool. On abdominal examination, there was gaseous distension with sluggish bowel sounds. Digital rectal examination revealed the presence of soft stools. Plain erect and supine abdominal X-rays demonstrated diffusely dilated bowel loops and multiple air-fluid levels without a clear transition point (Figures 1A, 1B). Abdominal ultrasonography further confirmed these findings, showing dilated bowel loops with markedly reduced peristalsis, but no evidence of mechanical obstruction or free fluid.

The diagnosis of paralytic ileus was supported by both clinical and radiological findings. Clinically, the absence of colicky abdominal pain, the presence of soft stool on digital rectal examination, and the lack of exaggerated bowel sounds pointed away from a mechanical cause. Ultrasound imaging revealed diminished peristaltic activity, which is more consistent with paralytic ileus; in contrast, mechanical obstruction typically presents with initially increased or hyperactive peristalsis proximal to the obstruction. Additionally, the absence of a definitive transition point on abdominal radiographs further argued against mechanical obstruction. A CT scan was not performed, as the clinical context and imaging findings were adequately consistent with paralytic ileus and did not warrant further evaluation for mechanical obstruction.

The patient was managed conservatively. To support gastrointestinal recovery, he was kept nil per oral, received intravenous fluids to maintain hydration and electrolyte balance, and underwent nasogastric tube decompression to relieve gastric distension and vomiting. Pain was controlled with the continuation of the same analgesics.

Over the next 48 hours, the patient’s symptoms gradually improved. His abdominal distension reduced, bowel sounds returned, and he started passing flatus, indicating resolution of ileus. The nasogastric tube was removed, and oral intake was gradually resumed. His urinary catheter was removed after 48 hours, and he was able to void spontaneously without difficulty. With complete clinical recovery, he was discharged in stable condition.

## Discussion

In the coastal waters of Sri Lanka, the observed jellyfish species include the lion’s mane jellyfish, the Portuguese man o’ war, and the box jellyfish [2]. Jellyfish stings can cause a wide spectrum of symptoms due to the release of venomous toxins, which contain a mixture of cytolytic pore-forming toxins, neurotoxins with activity at fast sodium and inwardly rectifying potassium channels, and nonprotein bioactive components [3,4]. The roles and impact of these toxins in clinical envenomation are still being explored and remain not fully understood. The clinical presentation of a jellyfish sting can vary depending on factors such as the species of jellyfish, the patient's characteristics, the duration of exposure, and the area of skin affected [5]. Local envenomation symptoms typically include pain, paresthesia, intense burning, and a linear, red, papular rash or urticaria at the contact site [6]. Systemic reactions to jellyfish stings can lead to Irukandji syndrome, cardiorespiratory arrest, and anaphylaxis [5]. Irukandji syndrome is a severe and potentially fatal condition caused by certain species of box jellyfish. Irukandji syndrome causes symptoms resembling sympathetic overactivity that can develop within about 30 minutes after the sting, including tachycardia, elevated blood pressure, intense pain, muscle cramps, and life-threatening cardiac issues [7].

Few cases of paralytic ileus following jellyfish envenomation have been documented, making it a rare complication. Paralytic ileus is a condition characterized by a loss of motility in the gastrointestinal tract due to neuromuscular dysfunction [8]. This leads to a failure of peristaltic movements of the gastrointestinal tract, causing a functional obstruction. Ponampalam R reported a case of a 31-year-old tourist who developed abdominal distension, vomiting, and absence of bowel movements after a jellyfish sting, similar to our case. The patient was ultimately diagnosed with paralytic ileus and successfully managed with supportive care [9]. The mechanism by which jellyfish toxins lead to paralytic ileus remains uncertain, but it is believed that the neurotoxins may interfere with the normal autonomic nervous system regulation, which is crucial for gastrointestinal motility. To reinforce this statement, a case report of a box jellyfish sting described the occurrence of reversible parasympathetic dysautonomia [10]. Urinary retention in our patient can also be attributed to parasympathetic dysfunction. Hypothetically, jellyfish toxin may disrupt parasympathetic regulation by interfering with acetylcholine-mediated transmission or enteric nervous system function, leading to reduced peristalsis and paralytic ileus. Similarly, urinary retention may result from impaired detrusor muscle activity due to parasympathetic dysfunction. Although not yet confirmed in experimental studies, these mechanisms offer a plausible explanation for the autonomic effects observed. The rarity of autonomic manifestations such as paralytic ileus and urinary retention may reflect individual host susceptibility, genetic predisposition, or variability in toxin composition; however, due to limited experimental data, these remain uncertain. Further research is needed to elucidate the precise neurotoxic mechanisms involved.

In most instances, jellyfish stings are diagnosed clinically or identified by the patient based on exposure to waters where jellyfish are known to be present. However, in cases where the clinical presentation is unclear, a definitive diagnosis may be necessary. This can be achieved by examining nematocysts under a microscope [5].

After a sting occurs, avoid scratching, scraping, or any mechanical actions that could rupture the nematocysts [11]. Without directly touching the affected area, rinse it with seawater or vinegar [11,12]. Avoid using fresh water, as it may cause the nematocysts to discharge. For the rare case of paralytic ileus following jellyfish envenomation, management is supportive, as in our case. Most cases of paralytic ileus after jellyfish stings are self-limiting, as evidenced by the gradual improvement in gastrointestinal function and bladder function in our patient.

Prevention of jellyfish stings largely involves public awareness and safety measures, particularly in regions with high jellyfish prevalence. The findings of a randomized, placebo-controlled trial suggest that Safe Sea (Kibbutz Ketura, Israel: Nidaria Technology Ltd.), a topical jellyfish sting inhibitor, is effective in preventing jellyfish stings in natural marine environments [1]. For fishermen, education about the risk of jellyfish stings and the availability of prompt medical care can significantly reduce the chances of serious complications.

## Conclusions

While jellyfish envenomation is typically associated with local symptoms such as pain and itching, rare systemic complications like paralytic ileus and acute urinary retention can occur, as demonstrated in this case. This highlights the importance of clinical vigilance in managing jellyfish stings, recognizing potential neurotoxic effects, and ensuring timely supportive care. Additionally, a public health approach to prevention, including education on marine hazards, is crucial. Although uncommon, these complications should be considered in cases of severe envenomation to facilitate prompt diagnosis and appropriate management.
